# Robust and accurate digital measurement for HER2 amplification in HER2 equivocal breast cancer diagnosis

**DOI:** 10.1038/s41598-017-07176-x

**Published:** 2017-07-28

**Authors:** Yuefeng Wang, Julia Y. S. Tsang, Yongmei Cui, Ji Cui, Ying Lin, Songli Zhao, Patrick T. W. Law, Sai Yin Cheung, Enders K. O. Ng, Gary M. K. Tse, Zunfu Ke

**Affiliations:** 1 Department of Pathology, The First Affiliated Hospital, Sun Yat-sen University, Guangzhou, China; 2 Department of Anatomical and Cellular Pathology, Prince of Wales Hospital, The Chinese University of Hong Kong, Hong Kong, China; 3 Department of Gastrointestinal Surgery, The First Affiliated Hospital, Sun Yat-sen University, Guangzhou, China; 4 Breast Disease Center, The First Affiliated Hospital, Sun Yat-Sen University, Guangzhou, China; 5 Department of Pathology, Sanshui General Hospital, Foshan, China; 6 Pangenia Lifesciences Limited, Hong Kong, China; 7 Department of Pathology, Tuen Mun Hospital, Hong Kong, China

## Abstract

Currently, there are no recommended alternative assays for HER2 cases deemed equivocal by immunohistochemistry and fluorescent *in situ* hybridization. Digital PCR (ddPCR), a highly accurate method to determine DNA copy number, could be a robust alternative for clinical HER2 diagnostics. *HER2 and CEP17* copy numbers were quantified using two ddPCR platforms (QX200 and RainDrop) in 102 samples of invasive breast cancers. Compared to routine assays, ddPCR gave a sensitivity and specificity of 82.8% and 97.3% respectively, with a kappa value of 0.833 (p < 0.001). Moreover, the method proved to be robust as the results from two platforms was highly correlated (R^2^ = 0.91; Concordance rate = 97%; κ = 0.923, P < 0.001). Its performance was further tested on 114 HER2 equivocal cases in an independent validation cohort. 75% (21/28) of cases with HER2 amplification and 95% (82/86) of HER2 non-amplified case were classified as positive and negative by ddPCR respectively (κ = 0.709, P < 0.001). Notably, in the HER2 amplified cases, a lower percentage of HER2 positive cells could be related to the discordant results. Altogether, ddPCR is a robust alternative for clinical HER2 diagnostics. However, intratumoral heterogeneity of HER2 status still pose a challenge for HER2 analysis by ddPCR.

## Introduction

Human epidermal growth factor receptor 2 (HER2) has been used as a predictive and prognostic marker in breast cancers. Its over-expression is predictive of response to HER2-targeted therapy, such as trastuzumab^1^. Evaluation of the HER2 status is now a standard of care for all primary breast cancers. The current techniques for HER2 testing include immunohistochemistry (IHC) for detection of protein expression and fluorescence *in situ* hybridization (FISH) for detection of gene amplification. Basing on the testing algorithm recommended by the American Society of Clinical Oncology (ASCO), in many laboratories, IHC is generally performed first. FISH is added if the result of IHC is equivocal^2^. Despite being widely used as a standard procedure, both tests have several limitations. Both are technically demanding, thus results may vary between different laboratories^3^. One of the underlying reasons is that they are easily affected by variations in testing conditions (such as fixation, reagent and assay protocol used^2^) thus compromising their reproducibility and accuracy. In addition, they are labor intensive and time consuming, especially for FISH. The manual and complicated procedure in FISH tests limit the number of patients that can be handled at any one time. Prolonged HER2 turn-around time was highlighted as a concern in the last national review in UK^4^. The manual scoring method could also be problematic. It is subjected to inter-observer variability, particularly in IHC which is done qualitatively rather than quantitatively^5, 6^. Another challenge is, in some cases, the test results can be indeterminate, either due to technical issues or the inherent features of the tumor. For instance, a proportion of the equivocal cases related to CEP17 aneusomy are due to focal amplification of the reference region rather than true polysomy. Inclusion of multiple controls can improve the results in those cases^7^. Standard FISH test, however, enumerated only CEP17 signal; therefore, a more robust alternative test which can perform multiplex analysis and deliver accurate, reliable and timely results is highly desirable.

Digital PCR (dPCR) has been developed recently for absolute nucleic acid quantification and rare allele detection^8^. This technique is amenable to high throughput development and can be applied to FFPE tissue^9, 10^. It has also the power for multiplex analysis^11^. Additional controls/targets can be included and examined at the same time, allowing multitude of information to be obtained in a single run. The feasibility of dPCR in HER2 testing has been reported using frozen samples, FFPE samples and plasma DNA^9, 10, 12, 13^. A good concordance with the results from standard HER2 tests has been demonstrated. However, its performance in comparison to the standard techniques has not been well established. In these studies, only limited number of samples was included, thus precluding a more detailed and systematic analysis, particularly on HER2 equivocal cases. In this study, a systematic evaluation of the usefulness of dPCR in assessing HER2 status in breast cancers was performed. We have compared 2 different dPCR platforms. The accuracy of dPCR compared to the standard techniques was examined. Additionally, we have examined the usefulness of dPCR to determine HER2 amplification in IHC 2+ cases.

## Results

**Figure 1:**
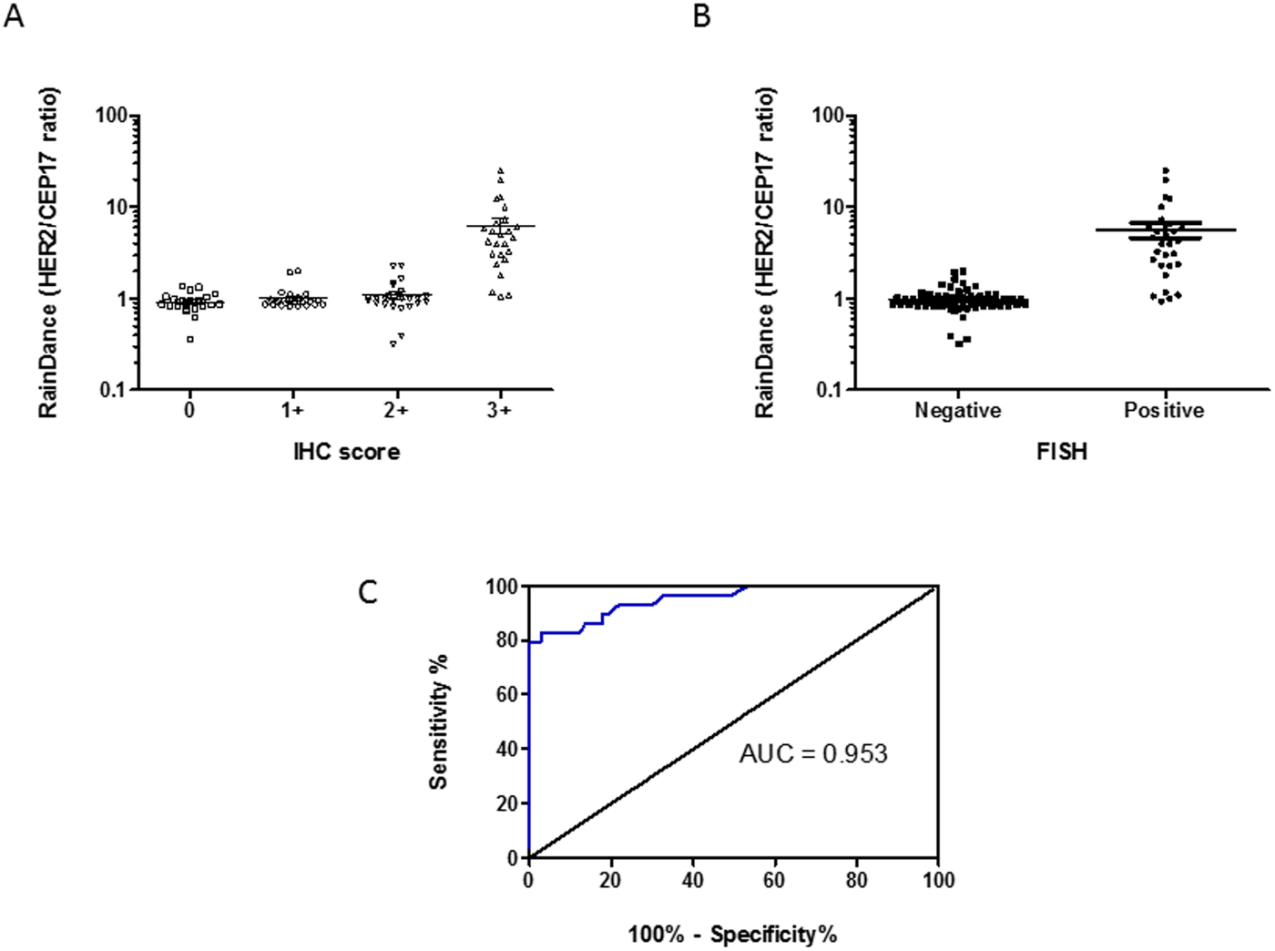
HER2:CEP17 ratio by ddPCR in the development cohort. (**A**) dot plot showing the HER2:CEP17 ratio with different IHC score and (**B**) HER2− and HER2+ status. (**C**) ROC curve to determine the optimal cutoff.

Of 102 cases of invasive breast cancers in the developing cohort, 25, 26, 26 and 25 cases were tested IHC 0, 1+ , 2+ , and 3+ respectively. There were 22 FISH negative and four FISH positive cases among the IHC2+ samples. Based on the IHC and FISH data, 29 and 73 cases were HER2 positive and negative respectively. The results of RainDance Droplet Digital PCR (ddPCR) analysis for the development cohort were shown in Fig. 1. The median (range) ddPCR HER2:CEP17 ratio in patients with IHC 0, 1+ , 2+ and 3+ were 0.90 (0.36–1.36), 0.93 (0.82–2.00), 1.00 (0.32–2.30) and 4.79 (1.06–24.84) respectively (Table 1). There are significant differences for the ratio between IHC 0 to 2+ and 3+ cases (p > 0.001) (Fig. 1A). However, there was no difference between IHC 2+ and IHC 0 and 1+ . When HER2 status were defined with ddPCR and FISH, the ddPCR HER2:CEP17 ratio in patients with HER2 amplified tumor (median 4.08, range 0.93–24.84) was significantly elevated compared to HER2 non-amplified cases (median 0.93, range 0.32–2.00, P < 0.001) (Fig. 1B). Analysis of the development set using data from RainDance platform by ROC had an AUC 0.9528 (95% CI = 0.9076–0.9979, P < 0.0001) (Fig. 1C). Based on the result from the development set, an optimal cutoff of 1.72 which gave a sensitivity and specificity of 82.8% and 97.3% respectively was found (Table 1). Using this cutoff, a kappa value of 0.833 (p < 0.001) was observed.

**Table 1:** Performance of RainDance and Bio-Rad ddPCR platforms on detection of HER2 amplification.

	IHC		N	HER2:CEP17 ratio (range)	ddPCR HER2−	ddPCR HER2+	ddPCR HER2−	ddPCR HER2+
**Raindance**								
Cutoff					1.72			
HER2−	0+		25	0.90 (0.36–1.36)	25	0		
	1+		26	0.93 (0.82–2.00)	24	2		
	2+	All	26	1.00				
		FISH−	22	1.00 (0.32–1.64)	22	0		
HER2+		FISH+	4	1.65 (0.93–2.30)	2	2		
	3+		25	4.79 (1.06–24.84)	3	22		
					Sensitivity	82.8%		
					Specificity	97.3%		
					NPV	93.4%		
					PPV	92.3%		
					Accuracy	93.1%		
**Bio-Rad**								
Cutoff					1.72		1.62	
HER2−	0+		24	1.01 (0.41–1.40)	24	0	24	0
	1+		26	0.97 (0.75–2.39)	25	1	25	1
	2+	All	24	1.03				
		FISH −	20	1.05 (0.06–1.78)	19	1	19	1
HER2+		FISH +	4	1.21 (1.07–2.62)	3	1	3	1
	3+		25	4.83 (1.11–27.3)	4	21	3	22
					Sensitivity	75.9%	Sensitivity	79.3%
					Specificity	97.0%	Specificity	97.0%
					NPV	90.7%	NPV	91.9%
					PPV	92.0%	PPV	92.2%
					Accuracy	91.0%	Accuracy	92.0%

We also compared the results of ddPCR from different platforms. Ninety nine samples were also assessed with Bio-Rad™ QX200™ Platform. HER2:CEP17 ratio measured by the two platforms showed good correlation with each other (R^2^ = 0.91) (Supplementary Figure S1). Bio-Rad platform by ROC had an AUC 0.9522 (95% CI = 0.9124–0.9920, P < 0.0001). A cutoff 1.62 gave a sensitivity and specificity of 79.3% and 97.0% respectively (Table 1). Despite an apparent discrepancy in their optimal cutoffs, if 1.72 cutoff was applied to the Bio-Rad data, only one case was re-classified to a different category. The concordance rate between the two platforms was 97%, with a Kappa value of 0.923 (P < 0.001). Discordant results were found in two HER2 negative cases which were classified as HER2+ by one but not the other system and one HER2+ case which was classified as HER2− by the Bio-Rad platform (Table 2). There were seven and eight discordant cases from IHC/FISH analysis using Raindance and Bio-Rad platforms respectively. There were two false positive and five false negative cases with the Raindance platform; and two false positive cases and six false negative cases with the Bio-Rad platform. Among those cases, one false positive and five false negative cases were seen with both platforms (Supplementary Table S1).

**Table 2:** Comparison of ddPCR results from different platforms.

		RainDance−	RainDance+	Total
HER2−	Bio-Rad−	67	1	68
	Bio-Rad+	1	1	2
	Total	68	2	
HER2+	Bio-Rad−	5	1	6
	Bio-Rad+	0	23	23
	Total	5	24	

**Figure 2:**
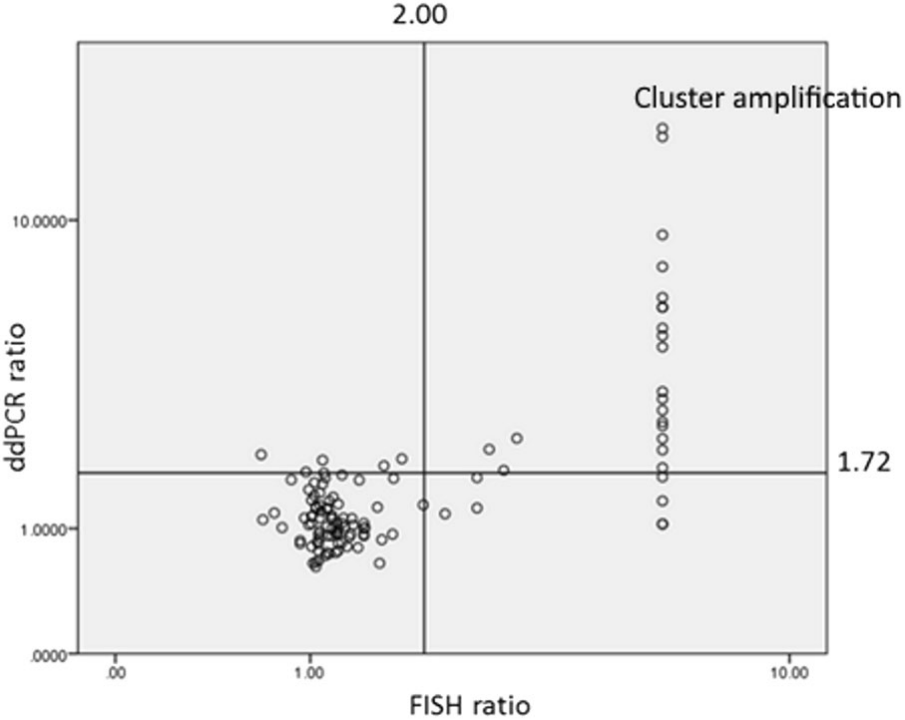
Correlation of HER2:CEP17 ratio between ddPCR and FISH in validation cohort.

The performance of ddPCR using the Raindance platform was assessed in an independent validation cohort enriched with IHC 2+ cases. Totally, 114 IHC 2+ cases were included in the cohort. Among them, there was 28 FISH positive cases in which six showed HER2 amplification as clusters and others with a median HER2:CEP17 ratio by FISH of 2.7 (range 2.23–3.17). The median HER2:CEP17 ratio determined by ddPCR was 1.04 (0.62–2.01) and 2.57 (1.05–17.29) for HER2 non-amplified and amplified cases respectively (Table 3). There was a significant difference in the ratio between the two groups (p < 0.001). There were 89 cases classified as HER2 non-amplified and 25 cases as HER2 amplified by ddPCR (Fig. 2). The number of concordant cases with FISH analysis were 82 and 21 for HER2 non-amplified and amplified cases respectively, with a kappa value of 0.709 (P < 0.001). The sensitivity and specificity by ddPCR for HER2 IHC2+ cases were 75.0% and 95.3% respectively (Table 3).

**Table 3:** Performance of RainDance platform in validation cohort.

IHC		N*	HER2:CEP17 ratio (range)		ddPCR HER2−	ddPCR HER2+
			FISH	ddPCR		
2+	All	114 (92)	1.15	1.14		
	FISH−	86 (86)	1.15 (0.68–1.99)	1.04 (0.62–2.01)	82	4
	FISH+	28 (6)	2.7 (2.23–3.17)	2.57 (1.05–17.29)	7	21
					Sensitivity	75.0%
					Specificity	95.3%
					NPV	92.1%
					PPV	84.0%
					Accuracy	89.5%

*(Number of cases with HER2:CEP17 ratio by FISH analysis).

In the HER2 non-amplified cases, there was no difference in tumor content and the percentage of HER2 positive cells between concordant and discordant cases. Of note, in the HER2 amplified cases, it appears that a lower percentage of HER2 positive cells (p = 0.055) was found in the discordant cases. No significant difference in the tumor content between concordant and discordant cases in the HER2 amplified tumors was observed (Table 4). We have further examined heterogeneity of HER2 amplification in those discordant cases. Both HER2 amplified and non-amplified regions could be found in those discordant cases (Supplementary Figure S2).

**Table 4:** Association of discordant results with tumor content.

		Concordance	Discordance	
**HER2−**				
% Tumor	Mean	23.9	10.0	0.211
	SD	17.5	0	
	Median	20	10	
	Range	1–70	10	
% HER2 positive cells	Mean	13.1	3.33	0.284
	SD	19.8	5.8	
	Median	5	1	
	Range	1–80	1–10	
**HER2+**				
% Tumor	Mean	12.2	17.5	0.260
	SD	11.5	15.1	
	Median	8.0	15	
	Range	1–40	5–50	
% HER2 positive cells	Mean	68.2	31.7	0.055
	SD	34.6	38.2	
	Median	80	10	
	Range	1–99	2–99	

## Discussion

Reliable and timely HER2 testing is of paramount importance in guiding decision regarding HER2 targeted therapy in breast cancer patients. The current guidelines recommend testing all invasive breast cancers for HER2, typically with IHC followed by FISH when IHC is equivocal^2^. Despite being routinely used for HER2 assessments, the suboptimal performance and subjectivity in interpretation of both assays remains problematic^14^. Moreover, IHC equivocal results were reported in up to a quarter of breast cancers^15^. Among them, approximately 20% of those cases would show HER2 amplification by reflex FISH analysis^16^. In about 4% of cases, an equivocal FISH result could be obtained^17^. For these cases with HER2 test result ultimately deemed to be equivocal, no alternative assays were recommended currently. In this study, we evaluated the feasibility of using ddPCR as an alternative method for HER2 testing. We found a good agreement between ddPCR with the standard methods in both our development and validation cohorts. Almost perfect agreement with standard assays were found in the development cohort. Moreover, the technique was robust and only minimal variations in results were obtained from different platforms. Equivocal cases resulting from standard testing is problematic in clinical laboratories. Previous analysis on ddPCR for HER2 testing did not address the usefulness of ddPCR as a reflex test for IHC equivocal cases^10, 13, 18, 19^. These studies used small cohorts and limited number of IHC equivocal cases in their analysis. In our validation cohort which comprised IHC equivocal cases, ddPCR successfully defined HER2 status for 102 cases out of 114 cases with an accuracy of 90% and a substantial agreement with the standard assays was observed. Our data indicated that ddPCR can complement the current standard techniques and represent a potential optional test for HER2 diagnosis.

Due to the important therapeutic implication, correct identification of patients with HER2 amplification is crucial. However, there are controversial issues involving cases with equivocal HER2 status. Assessment of HER2 gene status by dual color FISH can be troublesome sometimes^20^, particularly in cases with polysomy of chromosome 17. The current FISH assays used a control probe for chromosome 17 that hybridizes to a site near the centromere (CEP17). However, additional copies of CEP17 detected by FISH could be resulted from regional CEP17 gain/ amplification rather than polysomy. In fact, studies using FISH and microarray-based comparative genomic hybridization analysis demonstrated that whole chromosome 17 polysomy was uncommon in breast cancer (5.5% among all chromosome 17 polysomic cases detected by FISH)^20^. Cases having ratio < 2.0 with polysomic chromosome 17 indicated by FISH could have HER2 protein overexpression^21^. Recent studies suggested that additional reference probes could allow more accurate assessment of true HER2 amplification^7, 22^. The current ASCO/CAP guidelines also recommends to consider testing with an alternative chromosome 17 probe in patients with equivocal FISH results^2^. In this regard, ddPCR would be particularly suitable as a reflex test. Although in our current analysis, we have only used CEP17 as our reference so as to obtain a more comparable results with HER2 FISH test, an important aspect of ddPCR is its ability for multiplexing, thus multiple references could be included. The use of reference targets such as ELF5B, RPS27A and DCK has been suggested to be possible options^23^. Both Raindance and Bio-Rad platforms enable detection of two fluorophores. Based on implementation of different probe concentrations and ratios between both florescence labels, it is possible to perform higher multiplexing^24, 25^. With a higher number of droplets in Raindance, 5-plex assay has been reported for the platform while 3-plex assay was reported for the Bio-Rad platform^24^. The cost of multiplexing due to the additional primers and probes is minimal. Additionally, ddPCR is more amenable to automation and high throughput screening but with a shorter turnaround time, therefore providing a timelier reflex test result.

Despite the high concordance, discrepant results were found in a few cases by ddPCR. Unlike FISH in which analysis on 20 adjacent breast cancer cells was considered sufficient to establish a HER2-positive status, results from ddPCR without microdissection represent HER2 status across different parts of the tumor. Inclusion of considerable non-cancer stroma could lead to a dilution effect, thus potentially false negative findings. A recent study suggested that concurrent evaluation of tumor content ratio could allow an accurate determination of HER2 status by ddPCR even without microdissection^18^. Another possible cause could be intratumoral heterogeneity. Heterogeneity of HER2 gene amplification was recognized in at least 5% of breast cancers^26^. It poses a significant diagnostic challenge for IHC or FISH assessments in HER2 testing^27^, leading to an inaccurate determination of HER2-directed therapy suitability. In the current study, there was no difference in tumor cell content between the discordant and concordant cases, but a lower percentage of HER2 positive cells in the discordant cases. We have observed that differences in HER2 status in regions with different HER2 IHC staining. The findings indicated that false negativity was more related to the content of HER2 expressing cells rather than the actual tumor content, suggesting the impact of HER2 heterogeneity in the accuracy of ddPCR. Variation of HER2 status in sampling for ddPCR and FISH could also account for the false positive results. The reason of false positive discordance may also be due to the underestimation of the FISH signals due to incomplete hybridization of the FISH probes^28^. Moreover, genomic chromosomal instability or loss of the reference gene can result in overestimation of ddPCR signals; thus a high HER2 copy number^29^. High temperature processing during FFPE DNA extraction has been demonstrated to cause an overestimation of the target gene copy number due to denaturation and partition of single-stranded DNA into different droplets^30^. In addition, formaldehyde used in sample fixation could introduce nucleotide artifacts in DNA sequence^31^. Other possible reason could be due to off target amplification. Further optimization of the primers and probe could be critical to improve the current technique.

In summary, this is the first study showing that it is feasible to use ddPCR as a reflex test for HER2 equivocal cases. It would be considered as a potential alternative test for clinical HER2 diagnostics. Our work demonstrated that the robustness of HER2 status profiling using the ddPCR approach among different ddPCR platforms. The ddPCR assay is more objective and robust than the conventional techniques without the need of laborious and tedious workflow. It also allows the inclusion of multiple references for more accurate determination of chromosome 17 status. However, intratumoral heterogeneity of HER2 status still pose a challenge for HER2 analysis by ddPCR and further study is necessary.

## Methods

### Patient’s Data

Invasive breast cancer specimens were collected from three of the involved institutions. All specimens were formalin fixed, paraffin embedded, and routinely processed. The histological diagnoses were confirmed using WHO criteria (WHO book). H&E staining of sample will be reviewed from each case. One hundred and four cases collected from two of the institutes from China were used as development cohort for cutoff determination and platform comparison. One hundred and fourteen cases from the other two involved institutes from Hong Kong was used for validation. The percentage of tumour cells and HER2 positive cells in the area was recorded for the validation cohort. Research was approved by the Joint CUHK-NTEC Research Ethics Committee. All experiments were performed in accordance with relevant guidelines and regulations. All archival samples from pathology tissue bank were retrieved after its use for diagnosis retrospectively. The specimens were obtained in ethical manner and no potential harm will be caused to the patients. For patient confidentiality, all samples were coded by laboratory accession number and were non-identifiable. Therefore, the research could be permissible without consent.

### IHC analysis

IHC analysis was performed with PATHWAY anti-HER2 antibody (clone 4B5) (Ventana) according to manufacturer’s instruction with BenchMark XT automated slide-staining instrument (Ventana, Arizona, USA) and Ultraview Universal DAB Detection Kit (Ventana, Arizona, USA) after deparaffinization, rehydration and antigen retrieval. All slides were counterstained with hematoxylin. The slides were scored from 0 to 3+ according to ASCO guideline by at least two pathologists blinded to the clinical information and the results of other tests. Any discrepancies were resolved by reviewing at a multi-head microscope and a consensus reached. Tumour with 0 or 1+ (no staining or weak, incomplete membrane staining in any proportion of tumour cells) was considered negative. A score of 2+ (complete membrane staining that is non-uniform or weak but circumferential in distribution in ≥ 10%) was considered equivocal. A score of 3+ (uniform intense membrane staining of >10% tumour cells) was considered positive^2^.

### FISH analysis

FISH analysis was performed using the PathVysion HER2 DNA probe kit (Vysis) following the manufacturer’s protocol. Four micron FFPE tissue sections was used and evaluated for HER2 gene copy number with CEP17 control probe as reference. Fluorescence signals were visualized using a Zeiss Axioplan 2 microscope (Zeiss). The images were acquired with CCD camera and analysed with MetaSystem Isis Software (MetaSystem). At least 50 nuclei on 2 selected regions were counted. The scoring was carried out by the investigators blinded to the result of other tests. Additional cells were counted for ratio between 1.8 and 2.2. The HER2 amplification was defined if HER2/CEP17 ratio is ≥2 following the latest ASCO guideline^2^. CEP17 signals per nuclei ≥ 3 and < 1.35 were defined as polysomy^2^ and monsomy 17^32^ respectively.

### DNA extraction

DNA from FFPE samples was isolated with QIAamp® DNA FFPE Tissue Kit (QIAGEN, Les Ulis, France) according to the manufacturer’s instructions with modifications. Briefly, sections of FFPE samples were deparaffinized using repeated xylene and ethanol washes followed by overnight proteinase K digestion at 56 °C. After incubation at 90 °C for one hour to reverse the formaldehyde modification of nucleic acids, DNA was purified using the QIAamp MinElute column and eluted in 30 µl of DNase-free water. DNA quantity was measured by Qubit® 3.0 Fluorometer according to the manufacturer’s instructions.

### Digital PCR

Digital PCR was performed using the droplet digital PCR (ddPCR) method on the RainDrop (RainDance Technologies, Lexington, MA) and Bio-Rad QX200™ (Bio-Rad, Hercules, CA) platforms. Briefly, each PCR reaction was prepared in a 25 µl solution consisted of 1 X QuantiNova Master Mix (QIAGEN, Les Ulis, France) or 1 X ddPCR Supermix for probes (Bio-Rad, Hercules, CA), specific probes (5 µM), primers (40 µM) and 50 ng of FFPE DNA. Droplets (~4,000,000/reaction for RainDance and ∼20,000/reaction for Bio-Rad) were generated on the RainDrop Source or Bio-Rad QX200™ droplet generator following the manufacturer’s instructions. (HER2: probe 5′-/56-FAM/TGT GCG AGG /ZEN/CAC CCA GCT CT/3IABkFQ/-3′; forward primer 5′-CTC ATC GCT CAC AAC CAA GT-3′ and reverse primer 5′-CAG GGC ATA GTT GTC CTC AA -3′, CEP17: Raindance probe 5′-/5TET/ACG TGC TGC /ZEN/AAT AGG CGG TTG CCT A/3IABkFQ/-3′ or Bio-Rad probe 5′-/5HEX/ACG TGC TGC /ZEN/AAT AGG CGG TTG CCT A/3IABkFQ/-3′; forward primer 5′-GCT GAT GAT CAT AAA GCC ACA GGT A -3′ and reverse primer 5′-CTG GTG CTC AGG CAG TGC -3′). Emulsified PCR reactions were carried out in heated lid thermal cycler (S1000™ Thermal Cycler, Bio-Rad), starting with 2 min initial denaturation at 95 °C, followed by 44 cycles of 95 °C, 5 s and 60 °C, 20 s (using a 0.5 °C/min ramp rate) and a final hold at 98 °C for 10 min. PCR product was processed for signal detection. The samples were read on a RainDrop Sense instrument or Bio-Rad QX200™ droplet reader accordingly. Data were then analyzed using the RainDrop Analyst data analysis software or QuantaSoft™ software from Bio-Rad. The number of droplets corresponds to the HER2 (FAM)-positive cluster and CEP17 (TET/HEX)-positive cluster were counted. The HER2:CEP17 ratio was determined either by the exact droplet counts in Raindrop data or by calculating the copies per droplet from the Poisson distribution in Bio-Rad data. Samples with minimum of 0.0005% Raindance positive droplets^33^ or 0.01% Bio-Rad positive droplets^34^ were included in the calculation and those samples with positive droplets number lower than the lower detection limit were excluded. The performance of the platform has been evaluated using breast cancer cell lines with known HER2 status (Supplementary Figure S3).

### Statistical analysis

All analyses were performed using SPSS software (version 23) or Prism 5 (version 5.03). For cutoff determination, the development cohort was analyzed with a receiver operator curve (ROC). The concordance between different tests analyzed by determining the kappa coefficient (95% CI) using the kappa test. Kappa coefficient < 0.2 was considered poor, 0.21–0.40 fair, 0.41–0.60 moderate, 0.61–0.80 substantial, and 0.81–1 almost perfect agreement. Tumor content in concordance and discordance was compared using Mann-whitney test. Correlation was calculated with Spearman’s correlation analysis. *P* value < 0.05 was considered statistically significant.

## Electronic supplementary material

Supplementary Information
