# Supplementary material for: Robust and accurate digital measurement for HER2 amplification in HER2 equivocal breast cancer diagnosis
Source: Sci Rep. 2017 Jul 28;7:6752. doi: 10.1038/s41598-017-07176-x (PMC5533703; doi:10.1038/s41598-017-07176-x)
Supplement: Supplementary file 1 — Supplementary Information [file 41598_2017_7176_MOESM1_ESM.pdf]

Supplementary data

Robust and accurate digital measurement for HER2 amplification in HER2 equivocal breast cancer diagnosis

Running Title: digital PCR in HER2 equivocal cases

Yuefeng Wang[1#], Julia YS Tsang[2#], Yongmei Cui[1#], Ji Cui[3#], Ying Lin[4], Songli Zhao[1,5], Patrick TW Law[6], Sai Yin Cheung[7], Enders KO Ng[6], Gary MK Tse[2*], Zunfu Ke[1*]

[1]Department of Pathology, The First Affiliated Hospital, Sun Yat-sen University, Guangzhou, China
[2]Department of Anatomical and Cellular Pathology, Prince of Wales Hospital, The Chinese University of Hong Kong, Hong Kong
[3]Department of Gastrointestinal Surgery, The First Affiliated Hospital, Sun Yat-sen University, Guangzhou, China
[4]Breast Disease Center, The First Affiliated Hospital, Sun Yat-Sen University, Guangzhou, China
[5]Department of Pathology, Sanshui General Hospital, Foshan, China
[6]Pangenia Lifesciences Limited, Hong Kong
[7]Department of Pathology, Tuen Mun Hospital, Hong Kong

[#] Co-authorship: These authors contributed equally to this work.

* Co-correspondence

Table S1 Summary of ddPCR and FISH discordant cases

**Case no**	**IHC**	**FISH**	**RainDance**		**Bio-Rad**	
			HER2:CEP17 ratio	status	HER2:CEP17 ratio	status
**30**	1+	Negative	1.93	+	0.93	-
**18**	2+	Negative	1.48	-	1.78	+
84	**1+**	**Negative**	**2.00**	**+**	**2.40**	**+**
53	**2+**	**positive**	**0.93**	**-**	**1.07**	**-**
7	**2+**	**positive**	**1.00**	**-**	**1.10**	**-**
38	**3+**	**positive**	**1.06**	**-**	**1.11**	**-**
34	**3+**	**positive**	**1.10**	**-**	**1.17**	**-**
32	**3+**	**positive**	**1.17**	**-**	**1.25**	**-**
**106**	2+	positive	2.29	+	1.31	-

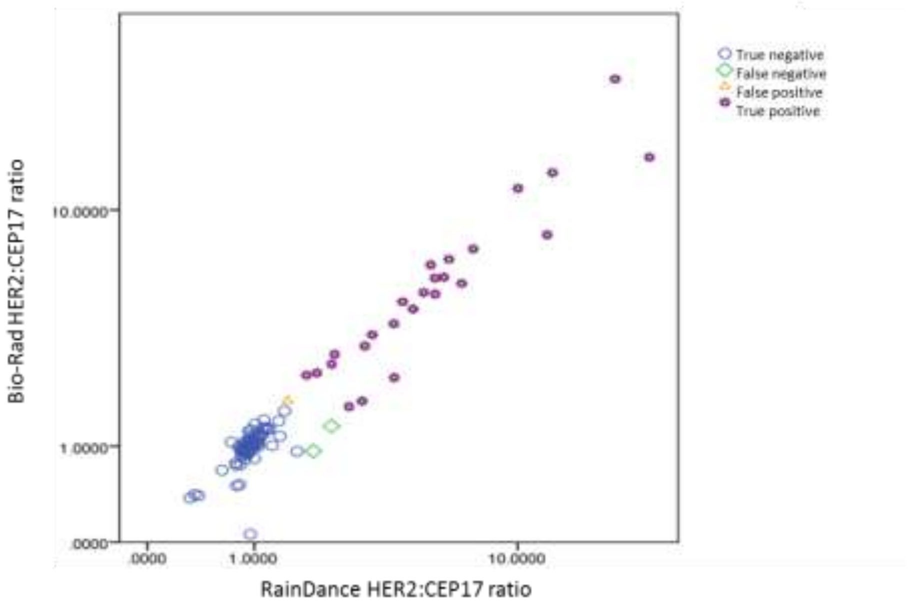

Figure S1 Correlation of HER2:CEP17 ratio determined by different ddPCR platforms.

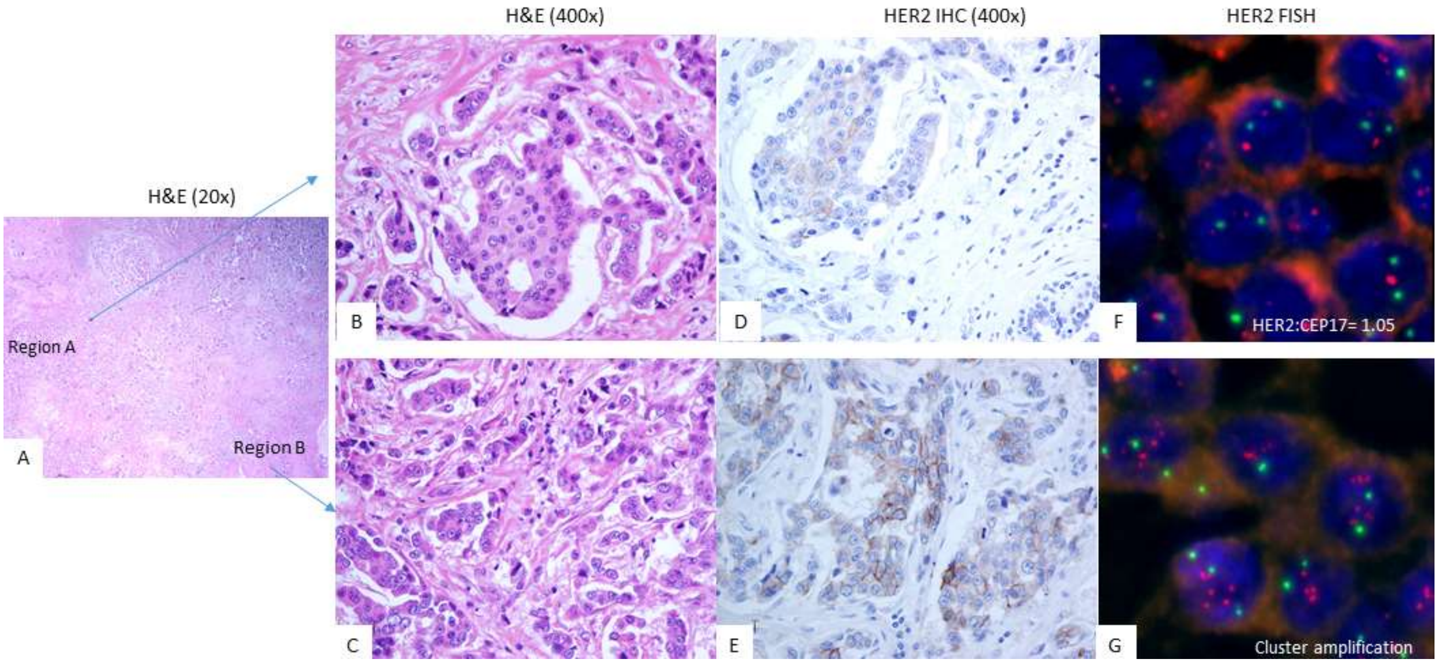

Figure S2 Heterogeneity of HER2 gene amplification in the discordant case. H&E of selected discordant case (A, B and C). HER2 non-amplified (D and F) and amplified (E and G) regions of the discordant case analysed by IHC (D and E) and FISH (F and G). Red and green signals showed HER2 and CEP17 copy numbers respectively.

A

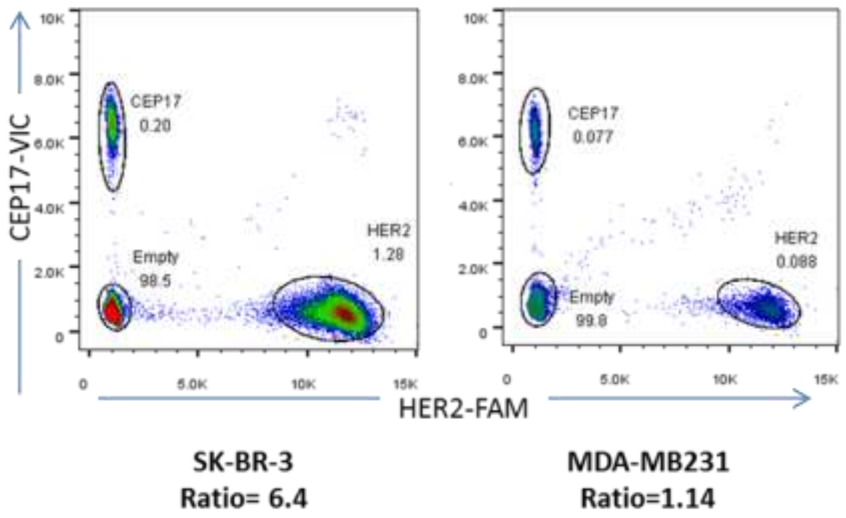

B

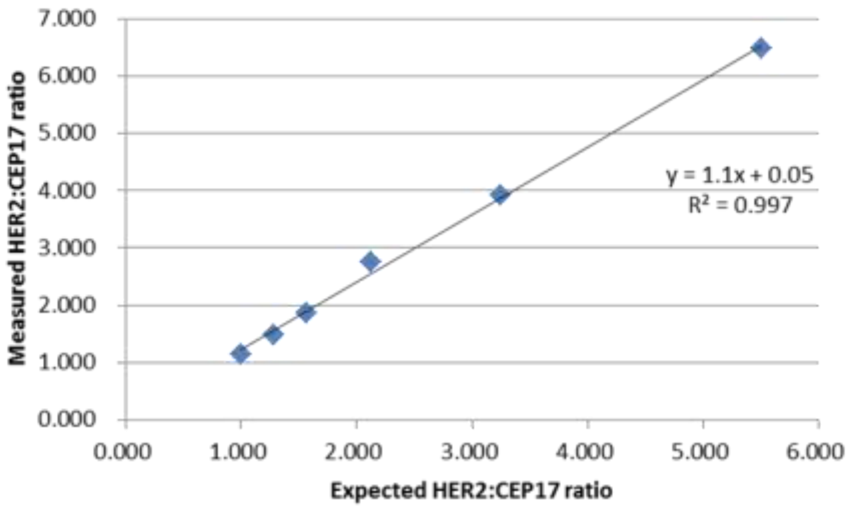

Figure S3 HER2 status of SK-BR-3 and MDA-MB231 determined by ddPCR. (A) Droplet digital plots showing HER2 and CEP17 counts and the number annotated the percentage of count in total droplet. (B) Determination of HER2:CEP17 ratio using SK-BR-3 DNA spiked into different ratio of MDA-MB231 DNA.
